# Socio-semantic networks as mutualistic networks

**DOI:** 10.1038/s41598-022-05743-5

**Published:** 2022-02-03

**Authors:** Jonathan St-Onge, Louis Renaud-Desjardins, Pierre Mongeau, Johanne Saint-Charles

**Affiliations:** 1 University of Quebec at Montreal, Montreal, H2X 3S1 Canada; 2 BIN, CIRST, Montreal, H2X 3R9 Canada

**Keywords:** Computational science, Scientific data, Cultural evolution

## Abstract

Several studies have shown that discourse and social relationships are intertwined and co-evolve. However, we lack theoretical models to explain the phenomenon. Inspired by recent work in ecology, we propose to model socio-semantic networks as an interaction between two intermingled data generating processes: a social community process and a document-based process. We consider the link between semantic and social ties as analogous to the interactions found in pollination networks whereby agents visit hidden topics in a similar way that insects visit specific plants for pollination. We use the ENRON socio-semantic email network to investigate if it exhibits properties that characterize mutualistic networks, namely moderate connectance, heterogeneous degree distribution, moderate modularity and high nestedness. To do so, we build a plant-pollinator matrix where “insect species” are communities detected via block modelling, “plant species” are latent topics detected with topic modelling, and the interaction between the two is the total number of visits a community makes to specific topics. Our results show that the ENRON socio-semantic interaction matrix respects the aforementioned criteria of mutualism paving the way for the development of a relevant framework to better understand the dynamic of human socio-semantic interactions.

## Introduction

Humans are fundamentally reliant on a rich web of mutually influencing social relationships and discourse forming complex socio-semantic networks^1–3^. Numerous studies have worked to elicit the processes and mechanisms behind this association, and many have called for the necessity to better model the co-evolution between social and semantic networks^1,4–8^. Inspired by the ecological literature and following on recent work done by Borge-Holthoefer et al.^9^, this paper answers this call by exploring the analogy that the interaction between human social relationships and discourse can be characterized as a mutualist network.

For decades now, studies in the field of socio-semantic networks have encompassed both social relationships and discourse elements, considered as representative of meaning, cognition, or culture^10–12^. A socio-semantic network can be understood as a network composed of connected entities (usually individuals or groups) and elements of discourse (words, concepts, sentences), called nodes. At its most basic expression, it is a two-mode network of entities linked by the elements of discourse they share. This two-mode network is often projected in a one-mode network in which the relationships between entities are the elements of discourse they have in common: the more they have in common, the stronger their tie. In other words, this projection seeks to uncover the structure of shared meanings between entities^13^. A more complex and quite frequent construction of such networks is the addition of social relationships such as friendship or work relation^14^, influence^15^, co-citations or scientific collaborations^1,16^ or twitter exchanges^17^. These studies have shown clear connections between the realm of the social and the semantic, whose connections allow for the identification of epistemic communities formed by connected agents sharing a set of discourse elements^18,19^. Nonetheless, we are still in need of frameworks to better understand the underlying drivers of this connection, and, notably to answer “concrete and contemporary questions on the existence of fragmentation and of possibly reinforcing socio-semantic clusters, often denoted as echo chambers”, in online public spaces^4,20^.

This issue connects with the homophily/contagion debate around information diffusion and adoption. This debate occurs around two competitive models aiming to explain the “assortative mixing and temporal clustering of behaviors among linked nodes”^21^, p. 21544: the homophily model^22^, for which sociodemographic similarity between agents leads to the development of social relationships, and the influence/contagion model^23,24^, where social relationships lead to the adoption of new information through a process of social influence. The grouping of people according to their interests for a common subject within online communities illustrates a semantic homophily phenomenon^1^ while semantic contagion would happen when nodes influence one another^25^. As illustration, the radicalization of a person’s political or religious positions could be explained by the relationships he or she maintains.

The concept of mutualist networks developed by ecologists offers a possibility to reframe the question of the primacy between social relations and the content of exchanges. A mutualist network is a two-mode, or bipartite, network that describes several species interacting in a mutually beneficial way^26^. In ecology, these networks embody two key advances in mutualistic thinking.

At first, mutualistic studies were unilateral, in the sense that investigators focused on a particular species of interest^27^. For instance, ecologists were assessing the role of animals in the life history of plants rather than focusing on the links between them. Adopting a more interactive lens, ecologists then focused on species-specific patterns (how one animal species of pollinator, the “visiting species” is highly adapted to a species of flower, the “visited “species). More recently, using a network approach, ecologists brought to the fore species interaction based on the idea of mutualism globally defined as interactions where both species derive benefit^26^.

Studies based on empirical observations of mutualism have revealed recurrent network patterns of these many-to-many interactions^12,13^. In a synthesis of these studies, Fernanda Valdovinos^28,29^ highlighted five structural properties shared by mutualistic networks:

### Moderate connectance

The connectance (C) of a network is the fraction of potential interactions that are realized. In mutualist networks, connectance tends to be relatively low (C < 0.3), which means that most of the links among potential mutualist partners do not take place^30^. This came as a surprise for ecologists since earlier studies commonly thought that an increase in connectance should be positively correlated with network stability, so that we should see high connectance in nature. Thus, moderate connectance has become a phenomenon of interest, alluding to key mechanisms at work in mutualist networks such as adaptive foraging^31^.

### Ratio visiting and visited species

According to Valdovinos most mutualist networks tend to have a greater number of animals (visiting “species”) than of plant species (visited “species”)^29^.

### Heterogenous degree distribution

Mutualistic networks are expected to have a low proportion of generalists compared to specialists leading to a long-tail degree distribution (for example, a truncated power law), which in turn suggests that most relationships are monopolized by only a few species (few generalists for many specialized species).

### Nestedness

Originally used in the field of biogeography, nestedness in plant–pollinator networks is the tendency of specialist species to pollinate the same subset of plants as generalist species. In other words, in a nested network most specialist species tend to establish specific relationships with a subset of the flowers pollinated by generalist species. Bastola^32^ has shown that nestedness is key for ecosystems primarily because it promotes their biodiversity by minimizing competition among species.

### Moderate degree of modularity

Modules are aggregated sets of interacting species, or clusters, that tend to interact more frequently within the cluster than with other clusters. Although there has been a debate on the degree of interactions that mutualistic partners have exclusively within modules (e.g. so-called compartments), there is a consensus that there is at least some degree of modularization in mutualistic networks^33^, p. 573. Ecologists have been particularly interested in modularity because it is thought to promote the stability of ecosystems because modules contain perturbation within them, thereby limiting consequences. Modularity has been shown to positively correlate with the degree of specialization of species in the network^34^.

We note that each criterion by itself is not sufficient. They must be understood in the context of each other. Only when taken together are these criteria sufficient to distinguish mutualist networks from other types of networks, such as the food web. In her review article, Valdovinos goes through how thee criteria lead to qualitative predictions, which have been either tested or are in need of being tested with empirical data^29^. These qualitative predictions seek to test the hypothesized mechanisms of mutualisms, which would be different in true ecological networks and socio-semantic networks.

To study socio-semantic networks as mutualistic networks, we construct an interaction matrix of the infamous ENRON email network. The ENRON corpus suits our need because it presents both a social and a textual dimension in the form of email exchanges and the content of these emails, respectively. We identify the social and the semantic as distinct entities in terms of a bipartite graph, thereby lending itself easily to the mutualistic analogy. We assume the following; (i) agents are our bees, thus social communities are our species, (ii) topics are species of flowers, and (iii) there is an interaction when a group member visits a topic.

Although we follow Borge-Holthofer et al.^9^ by casting socio-semantic interactions as mutualistic, we part ways on how we model the social and the semantic. Borge-Holthofer et al. use hashtags and users in Twitter network data as a proxy for discourses and social entities, respectively. This has the advantage of being a more concrete notion of a socio-semantic system, but one that is limited to Twitter. In contrast, we use topic modeling and social communities as our models for discourses and social entities. Our methodology is thus more general as it can be deployed in any context where we have social and text data. This generalization of the model is based on two premises, namely that discourses take the form of topics and that communities are well approximated by block modeling. Our work also goes further in the mutualist analogy, as we are not only looking for the modularity and nestedness of the social network structure extracted from ENRON’s email exchanges, we aim to test the hypothesis that a socio-semantic network shares many of the most important structural properties of plant–pollinator networks.

## Results

We investigate the socio-semantic network of ENRON from March 1999 to February 2002 (see Fig. 1 in Supplementary Materials for a time series of the number of email exchanges). The social dimension of ENRON corresponds to email exchanges between pairs of core employees, that is, employees who saw their mailboxes publicly released by the Federal Energy Regulatory Commission (FERC). The semantic dimension of ENRON is composed of the original message of emails. By original messages, we mean the content authored by the sender (therefore excluding forwarded emails or email threads). We extensively preprocess the semantic data to avoid redundancy and thus bias our algorithm towards repeated content (see Sect. II of the Supplementary Materials for more details).

**Figure 1:**
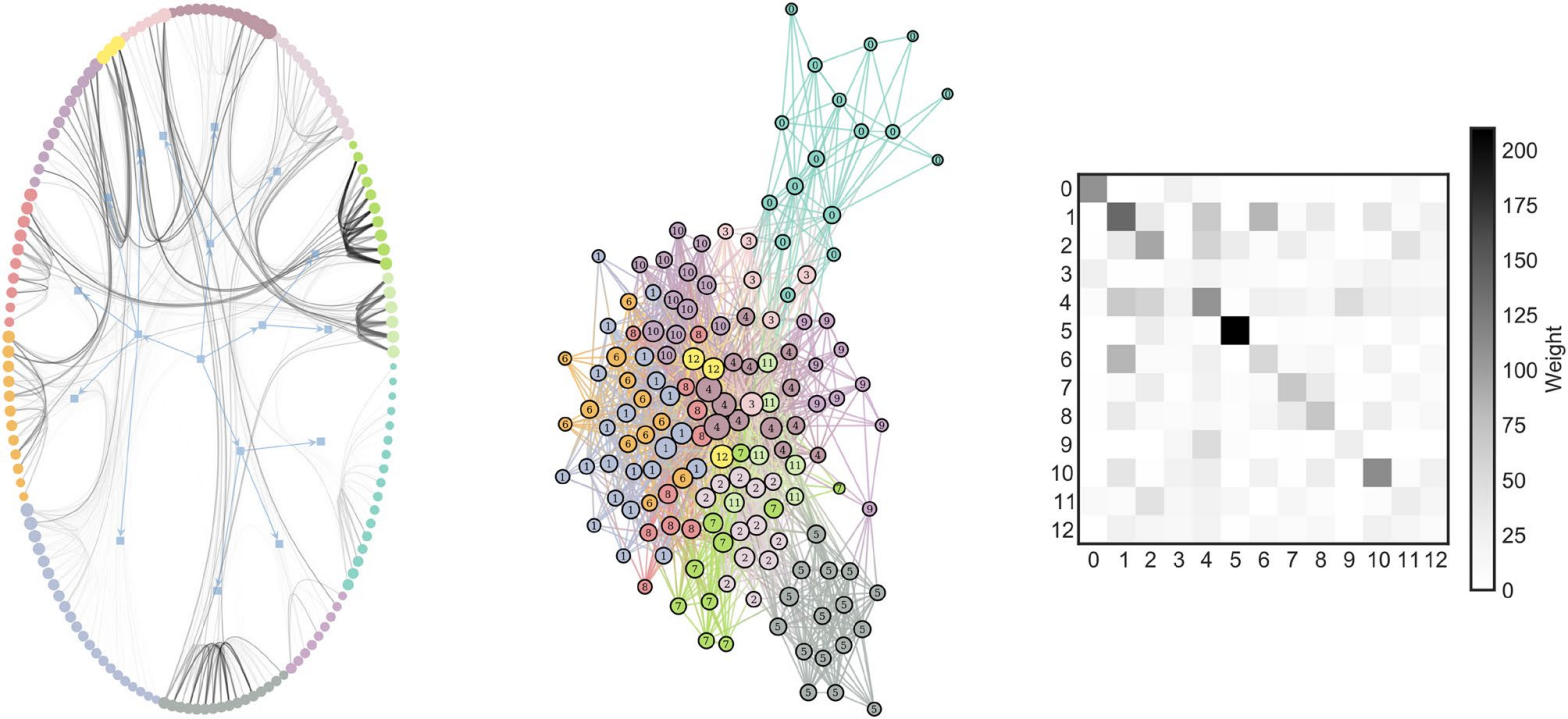
ENRON social network divided into 13 communities. On the left, is a hierarchical representation of the ENRON social network in which the agents are aligned on the periphery of the circle. This highlights, for example, that the 3 individuals from community 12 exchange massively with other communities. This representation is hierarchical as we can see higher-order groups in the form of blue blocks. In the middle and on the right, we have the most concrete level of the hierarchy represented as a single layer network and block matrix, respectively.

Using hierarchical Stochastic Block Models with a geometric distribution to model edge weights^35^, we find that the ENRON social network has 13 communities, ranging from 3 employees up to 28 (Fig. 1, left). The smallest community, community 12 (in yellow), is noteworthy as it is well connected to many other communities. We know from previous work that these 3 employees, Louise Kitchen, Philip Allen, and Mike Grigsby, often rank at the top of the different centrality measures^36^. We note the presence of a main component (Fig. 1, center), which surround these 3 individuals, with a moderately populated periphery composed of community 4 (in brown) and 0 (in teal). From the block matrix (Fig. 1, right), we can see that the community detection algorithm found blocks on the diagonal, where users within the community discussed more within themselves than across communities. This is especially true for users in community 4 who mostly discuss among themselves.

Now that we have introduced the social dimension of the ENRON network, we turn to the semantic dimension. We use the Correlated Topic Model (CTM)^37^ to identify the topics underlying the email exchanges among the ENRON employees. After model validation, we found that 40 topics give us quality topics that are neither too general nor too concrete. To assess the CTM output, we first look at the most common topics, as given by the parameter $\gamma$ (see Fig. S1). We interpret each topic by looking both at the most probable and the most frequent terms, as given by the $\beta$ parameter and FREX score, respectively.

We note that the most important topics are mainly general topics related to the English language (topic 1: know, can, let, thanks, please), or administrative queries (topic 23: please, call, attached, draft, review). Then we have topics associated with the fall of ENRON such as topic 16, related to the Federal Energy Regulatory Commission (FERC), and topic 38, related to California businesses. We also find topics that are recurrent in modeling topics from the ENRON corpus, such as topic 21, which is related to the fantasy league that we know occurred between employees. A complete list of topics with their most prevalent words can be found in the Supplementary Materials.

**Figure 2:**
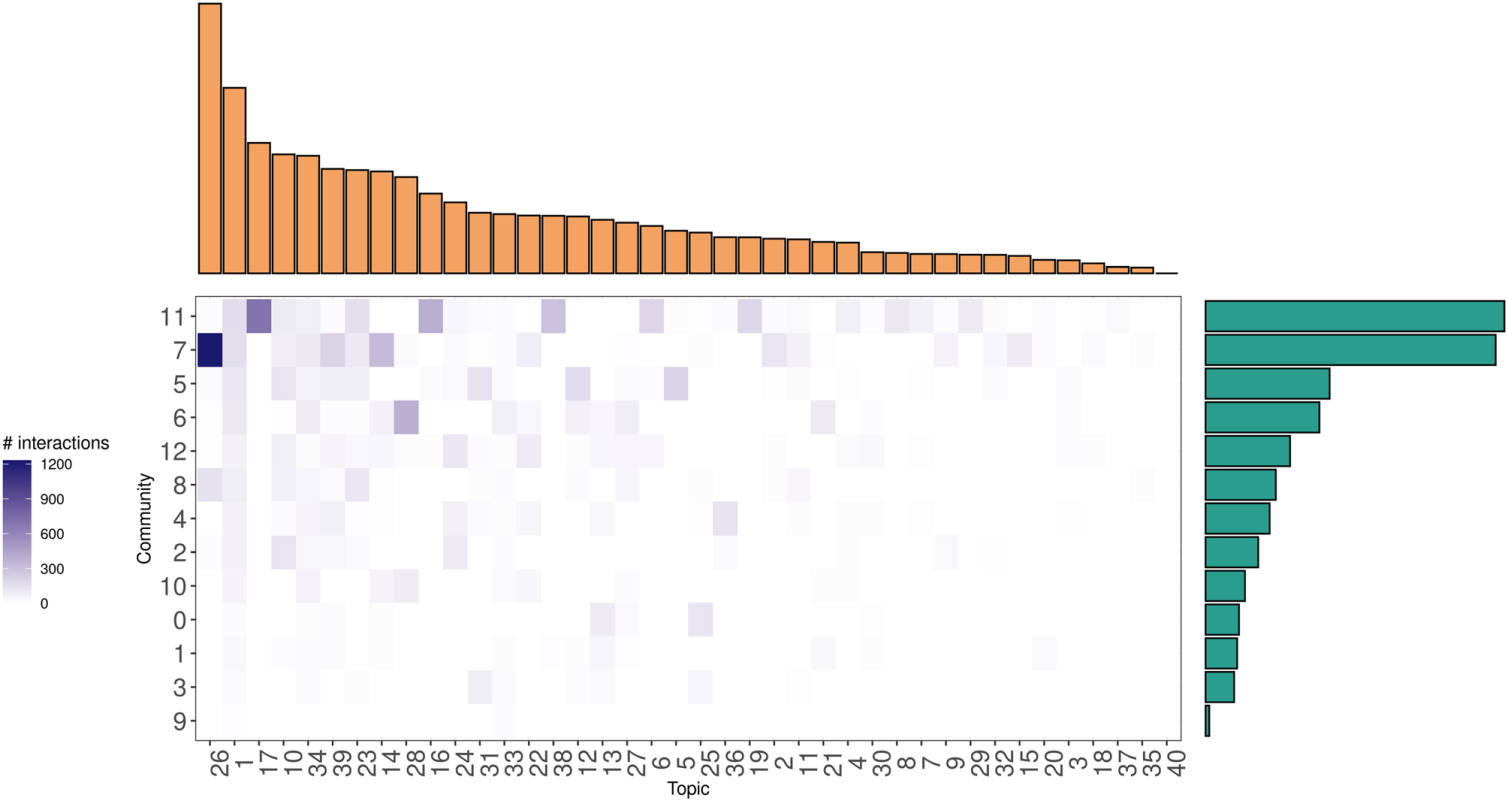
ENRON corpus as a mutualistic network. The interaction matrix is ordered such that most interactions occupy the upper left corner of the matrix. This arrangement is useful to see at a glance the degree of nestedness of the matrix. We note that both axes exhibit a long-tail distribution, which means that generalist communities and topics tend to interact together and that we have a lower proportion of generalists compared to specialists.

We finally bind together the social and semantic into an interaction matrix to assess their mutualistic tendencies. We can see in Fig. 2 that many elements that characterized a plant-pollinator matrix are present, with most of the interactions found in the upper left-corner of the matrix. We first note that the connectance of the ENRON interaction matrix is 0.389, a value we can consider moderated when compared to the 148 mutualistic networks available at the web-of-life ecological network database (see Supplementary Materials Sect. IV).

**Figure 3:**
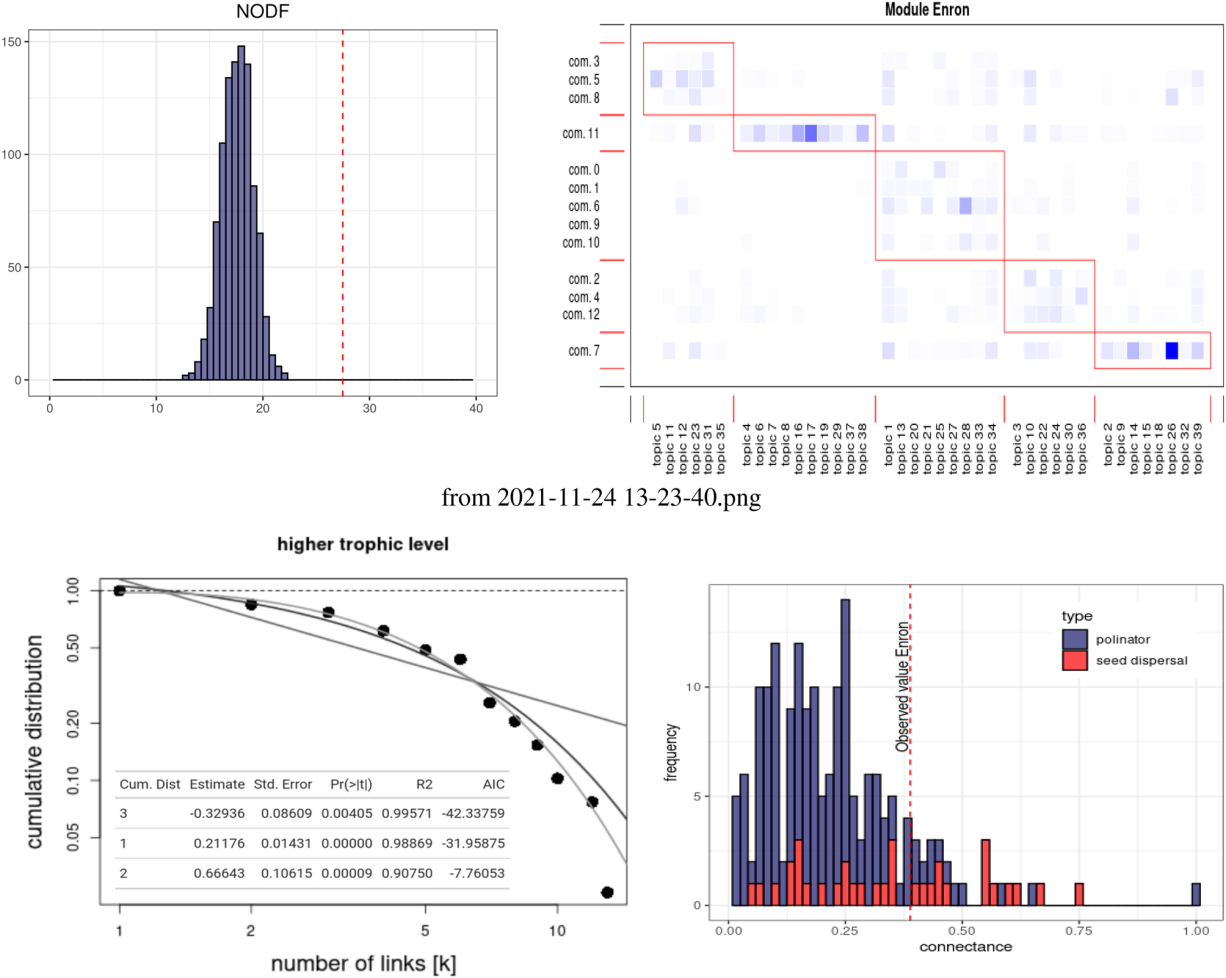
Summary null models. Top Left: The distribution is the output of patefield algorithm, in which we calculate the NODF value for each of the 2000 null models. The red dotted line represents the NODF value for the ENRON interaction matrix. Top-right: The matrix containing the modules in the ENRON network, as detected by the QuaBiMo algorithm^34^. Bottom left: The goodness of fit of various long-tail distributions to the ENRON degree distribution. The truncated power law is favored as indicated by the smaller AIC score. Bottom right: A comparison of observed connectance relative to true ecological networks.

The histograms on both axes correspond to the marginal totals of our two kinds of species. In the ecological literature, they are commonly interpreted as species abundance^33^. As in mutualistic networks, we can see that both marginal totals are dominated by few species, with most species living in the tails. We find that degree distribution is best approximated with a truncated power law, relative to a power law and the exponential distribution (see Fig. 3 bottom). Table of coefficients shows the different fit, where we can see that the Akaike Information Criterion (AIC) prefers the truncated power law. We can see that community 11 and 7 have the highest number of partners, while most other (specialists) communities live in the tail.

Regarding nestedness, the ENRON interaction matrix has a Nestedness Overlap and Decreasing Fill index (NODF) of 27.5. Since this value could only be due to the abundance of species, it is not in itself particularly significant. As is often the case in ecology, we compare the observed NODF value to a null model, here using the Patefield algorithm^38^ (see Fig. 3). With Patefield algorith, we shuffle individual counts while keeping both marginal totals fixed. In other words, the total number of topics and people in communities is fixed, but we randomize the number of visits from communities to topics. As such, Patefield algorithm disrupts both the social and the intra-topic correlations since this is the interaction beteen communities and topics that are shuffled during the randomization procedure. In each iteration, we calculate anew the NODF value. In our case, we shuffled 2000 null models. We find that the observed value is significantly greater than expected with our random procedure. Finally, we note that the matrix exhibits a modularity of 0.434. When compared to other plant-pollinator matrices, this value is also typical (see Supplementary Materials Sect. IV).

**Figure 4:**
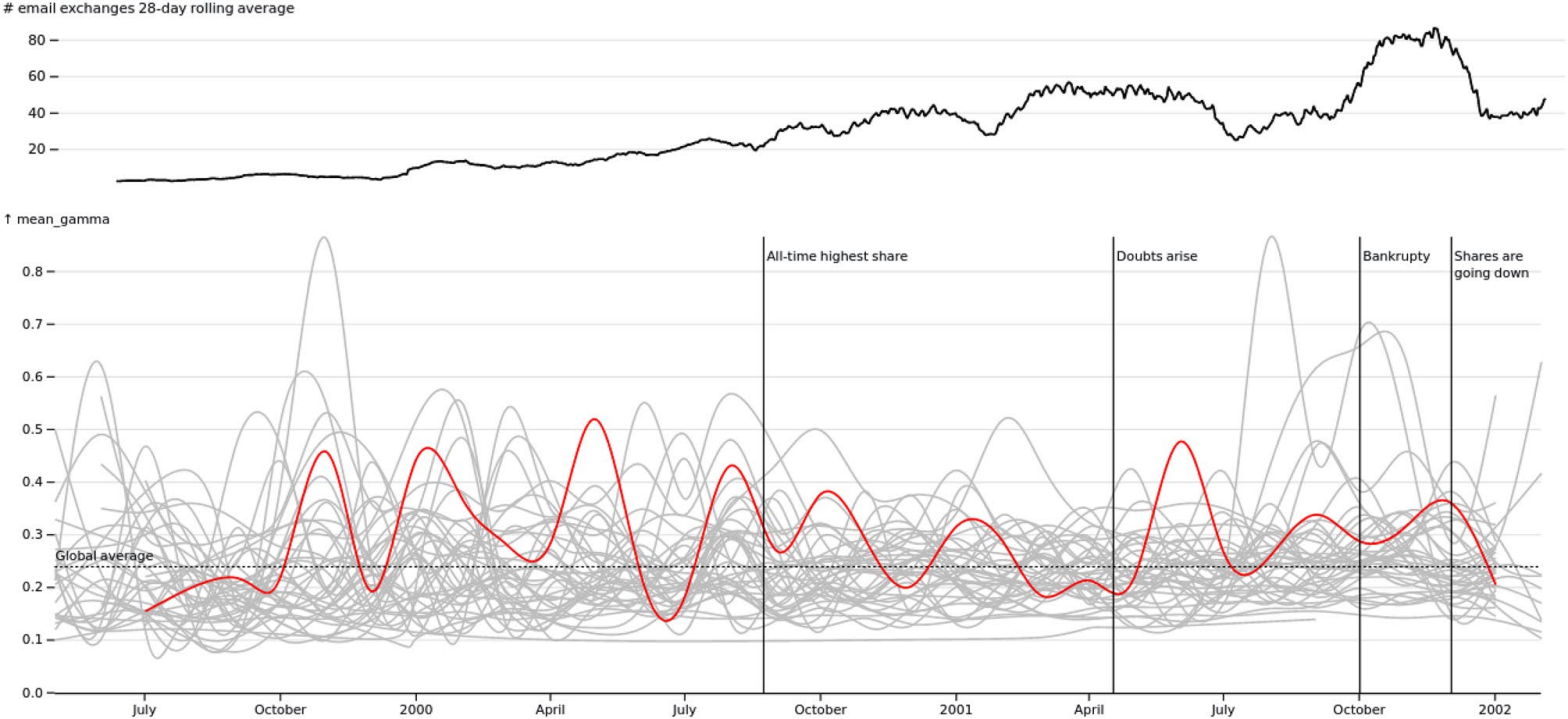
Evolution of topic 25 (mw, ercot, purchase, sale, firm, schedule, short, pge, sell), which is a specialized topic for community 6.

We now assess the interaction between communities and topics to explore how particular communities relate to specific ideas, and vice versa. Communities 6 and 0 are good examples of specialists as they mostly discuss topics 21 and 25, respectively. Our method offers the possibility to further investigate these relationships. In this case, topic 21 seems to be mainly related to entertainment (top words are free, site, draft, pick, football), while topic 25 is related to business discussions surrounding energy transactions (mw, ercot, purchase, sale, schedule). Based on available metadata, we can see that both communities are composed of employees and managers, which we might hypothesize were representative of specific departments (see Fig. 4).

We can contrast the above specialized interactions with topics visited by community 11 (pale green in Fig. 1), one of the most generalists and connected communities of the dataset (along with community 7). Although this community is generalist, it should be noted that it is not the most populated one. This fact is important because it is a recurring argument in ecology that the species abundance, and not the ecological process of mutualism, might explain the observed pattern. The community is formed of Mary C. Hain, Jeff Dasovich, James D. Steffes, Richard B. Sanders, Steven J. Kean, and Richard Shapiro. They discuss the well-known scandal of Enron with the state of California (topic 17 and 38, the so-called California electricity crisis) together with the FERC (topic 16), they also discuss legal meetings (topic 10), and eventually the ENRON bankruptcy (topic 11). Thinking in terms of mutualism, it is worth noting that both this community and community 7 have much higher weighted closeness and betweenness, meaning that if they were to be removed the network would be less stable (each department would be on their own, both socially and semantically). Interestingly enough, Mary C. Hain has subsequently been hired by the US government as an attorney who is acutely aware of fraudulent schemes, precisely because of her generalist role in the ENRON scandal.

Using available metadata, w.

## Discussion

The aim of our study was to explore the relevance of mutualism as a framework to help understand how the social and the semantic are linked in digital exchanges. We did this by analyzing the ENRON Corpus through the lens of an insect–plant pollination model. The insect species are deemed to be communities emerging from email exchanges while plant species are the topics detected in the content of the emails. A community visits a topic (like a bee visits a flower) when a community’s email content is related to that topic. By counting the number of visits from each community to each topic, we build a matrix of interactions. Based on the criteria put forth by Valdovinos, namely high nestedness, moderate connectance, heterogenous degree distribution, and moderate modularity, our results demonstrate that the ENRON socio-semantic networks can be characterized as a mutualist network.

What do we gain from this analogy? Studies bringing together the social and the semantic have shown how connected they are and postulated a co-evolutive phenomenon. The plant–animal relationship is thought of as a complex system that can be explained considering both local and global processes in a way analogous to the local discussions about specific topics and the global network of exchange in online socio-semantic networks. We have shown that the ENRON corpora can be understood with the lens of mutualism, above and beyond the idea of a co-influence of the social upon the semantic or vice versa.

Indeed, if our findings do not resolve the homophily-contagion debate, as this would require longitudinal data, it does offer another path to tread, more closely related to the idea of the co-evolution of the social and the semantic^39^. This is also of relevance given the difficulty of distinguishing between effects of homophily and contagion and hence to determine the primacy of one over the other^21^. By framing both the social and the semantic entities as species, one can start looking at how beneficial one is to the other rather than trying to answer the primacy question. From a longitudinal standpoint, questions would then resemble “when does a topic becomes beneficial for a community of entities?” or “When is a community no longer useful for a topic to survive and diffuse?” This is coherent also with what Borge-Holthoefer et al.^9^ proposed from their observation that “collective attention around a topic is reached when the user-meme network self-adapts from a modular to a nested structure”.

This change of perspective is also potentially fruitful for exploring phenomena of echo chambers and fragmentation on the web. In ecology, investigators have shown that the structure of interaction patterns is a key factor to explain biodiversity and ecosystem survival, going as far as to say that plant–animal mutualistic networks can be regarded as the architecture of biodiversity^40^. Can something similar be said about socio-semantic network, e.g., the mutualistic relation between the social and the semantic being the architecture of cultural diversity? If so, the joint study of communities and topics could prove useful to better understand echo chambers. Echo chambers are commonly defined as subsets of individuals who are primarily exposed to ideologies that agree with their own political leaning^41^. This has become a topic of great interest in recent years as social media is thought to facilitate their emergence. A prototypical case of echo chambers is the discussion surrounding “gun right users” and “gun control” in the United States, where two camps largely ignore one another in their Twitter interactions^42^.

In our analogy, echo chambers could be considered as compartments in ecology. That is, when a subset of species does not interact with other subsets^33,43^. Compartments imply strong modularity, while moderate modularity allows for some overlap between subsets (which can be observe in Fig. 3—top right). The general idea is that compartments act as a buffer against perturbations and thus increase the stability of ecosystem functioning^44^. In the context of online socio-semantic communities, high modularity may lead to echo chambers, social fragmentation, and conflict. On the practical side, interventions aiming at favouring cultural diversity online will be constrained by this high modularity, meaning that isolated groups might be more resilient to interventions that promote diversity of ideas.

From a social viewpoint, we would prefer to foster environments that facilitate mutualistic interactions rather than competition as this should increase the number of coexisting topics, which in turn could decrease polarization^42,45^. Therefore, seeing echo chambers through the mutualistic lens suggests new avenues of research as to why communities fail to entertain “national conversations”, and instead isolate themselves within their own set of beliefs^46^. A better understanding of the transition between these two modes of conversation could lead to more optimal strategies to counter polarizing states. A mutualistic perspective offers new directions to follow to prevent such outcomes. Borge-Holthoefer et al.^9^ have already shown a movement from high modularity to mutualism. How did this happen? What mechanisms have been in play to trigger this passage? These are interrogations whose answer may well come from adopting a mutualistic viewpoint.

Caution should be exercised in extending our analogy between cultural and biological systems. In many ways, cultural and biological systems are similar. Both are hierarchical complex systems with nonlinear interactions giving rise to a global structure which might not be expected from local behavior. But they also differ considerably at a closer look. Plants and pollinators have co-evolved over millennia. This biological co-evolution puts constraints on interactions that we do not find for cultural systems^47^. Also, some connections are simply not possible because of phenological or physical size constraints, what has been dubbed “forbidden links”^40^. Another point of great dissimilarity is the observation process. While ecologists build their models based on fieldwork observation, we have constructed our mutualistic networks based on the output of two latent variable models. An important consequence is that what we consider to be observed is sensitive to the assumptions built into our methods.

We also need to be aware that nestedness as a metric that captures the mutualist phenomenon has its critics. One convincing line of argument by Payrató-Borràs et al.^48^ is that tools to assess the significance of nestedness are not stringent enough to really distinguish underlying phenomena from entropic side effects. The take-home lesson of Payrató-Borràs’ article is that the disassortive structure, or heterogeneous distribution of degrees, implies greater entropy than non-disassortative structure, and as such could lead to the observed nested structures. One way to reconcile this fact with nestedness as an ecologically relevant metric is to recognize that the debate boils down to the choice of null models^49^. In any case, we leave this question for future work.

There are also technical limitations that we could overcome in further work. For example, how to best represent text remains an open challenge. In our case, the choice of the correct number of topics, at a given time window, is still highly problematic. It is hard to count species that have no materiality. Then, topics themselves might not be the best type of text representation, as the distributional semantic fails to represent higher-order structure and resolve words polysemy. Similar arguments apply for most community-detection methods.

Another area for further exploration would be the type of ties used to capture the social dimension. For ENRON the communities brought out by block modelling are based on observable email exchanges. In future developments, our exploration could be replicated with different types of ties. Although observed relationships online are limited to posts, tweets or email exchanges, it might be possible to infer the type of relationships from the text itself in a way similar as that proposed by Choi et al.^50^ who tried to find the presence of the 10 types of relationship proposed by Deri et al.^51^ typology in various corpora, including the ENRON dataset. With similar machine learning approaches, it might be possible to infer types of ties based on the various typologies of social relationships that have been studied in social network analysis^52–54^.

In the burgeoning field of socio-semantic analysis of digital exchanges, several other “variables” have been considered and have shown some explanatory power for the link between the social and the semantic. For instance, expert and contextual knowledge^1,55^, emotions^16^, actors’ characteristics such as personality, roles or status^56–59^ have been considered. These considerations could be reinterpreted under the lens of a mutualistic framework with novel interrogations around factors affecting the grouping of actors above and beyond the semantic and factors affecting the primacy of certain topics.

In closing, our work, as most work in this field, assumes the anthropocentric position that communities are the active species, while topics are the passive species even though we have more topics than communities while in ecology visiting species tend to be more numerous than visited species. We could have argued the opposite. As noted by Borge-Holthoefer et al.^9^, memes compete for the scarce resources that are their “hosts.” This brings to consideration the memetics framework^60^ and invites us to assume that there are good reasons to argue that topics visit communities of subjects. The main postulate of this theory is that ideas are like parasites that seek to survive and self-replicate. As all our tests work both ways, their results can also be interpreted both ways and the conclusion we draw would then depend on the theoretical posture adopted.

## Materials and methods

### Data

To test our hypothesis, we use the publicly available ENRON email network by Arne Hendrik Ruhe^61^. We only keep emails produced by the “core” employees, namely the 145 individuals whose mailboxes were published with the data in 2001. We investigate the email exchanges for the whole period of ENRON activity, that is, from March 1999 to February 2002 (see Fig. 4 top for a time series of the number of email exchanged). We extensively cleaned the dataset so that we extract only the original messages of the core employees. In doing so, we exclude all types of forward messages, email threads, or any other boilerplate text from the body of the text. We preprocessed the data as to focus on the original content of email exchanges. After cleaning, the dataset has 14,470 emails, for a total of 38,312 words (or tokens) and a vocabulary of 7234 unique words (or types).

Some emails such as forward emails have identical content, but different ids. Although they are redundant from a semantic point of view, they are distinct from a social perspective. Accordingly, we choose to count these emails as different exchanges for the social network, but not for the semantic network. Our social network is thus a weighted graph exchanges of email between pairs of core employees.

### The degree-corrected nested stochastic block model

From a Bayesian perspective, the goal of block modeling is to infer the posterior probability of node partition into B blocks, given that we observe an adjacency matrix $A_{ij}$^62^. To do so, the above generative process is translated into the following likelihood function:

$$\begin{aligned} P(A \mid \theta , w, b)=\prod _{i \le j} \frac{\theta _{i} \theta _{j} w_{b_{i} b_{j}}^{A_{i j}}}{A_{i j} !} e^{-\theta _{i} \theta _{j} w_{b_{i} b} j}, \end{aligned}$$

where the probabilities of observing pairs of edges in adjacency matrix A, given the parameters ,w, and b, are distributed according to a Poisson distribution. We can think of the likelihood function as the “forward direction”, e.g. given specific model configurations, what is the most likely data. Then, if we specify a prior probability over the parameters, we can use Bayes rule to go in the inverse direction, and infer the modular structure:

$$\begin{aligned} P(b \mid A)=\frac{P(A \mid \theta , w, b) P(b \mid \theta , w) P(\theta )}{P(A)}. \end{aligned}$$

This posterior distribution provides us with our desired values, that is, the membership of each individual within a group, given the observed data. The key components of this model is the interaction between the likelihood, $P(A|\theta ,w,b)$, and the prior over parameters, $P(\theta ,b) = P(b|\theta , w)P(\theta )$. As it is often the case, we can ignore the denominator, P(A), also called the evidence, as it does not depend on the parameters.

Whereas the single-layer SBM uses flat priors, the ndcSBM replaces noninformative priors by a hierarchy of priors and hyperpriors, which amounts to a nested SBM, where the groups themselves are clustered into groups, and the matrix *e* of edge counts are generated from another SBM, and so on^35^. In other words, instead of putting flat priors over the parameters of the models, Peixoto proposed to recursively use models to describe higher-order aspects of the model. These higher order aspects include the number of groups, their sizes, and the partition of nodes into them. The recursion of models is still motivated from the principle of maximum indifference, whereby the higher levels of our model hierarchy come from maximum entropy probability distributions (for more details, see Peixoto 2017).

### The correlated topic model

We can more precisely describe the CTM through its set of probabilistic assumptions that formulate the generative process. Following Blei and Lafferty^37^, we denote documents by $d_{1...D}$, and that there are n words indexed by position, $n_{1...N}$. We refer to word *n* in document *d* as $w_{n,d}$, which comes from a vocabulary of interest, $v_{1...V}$. Each topic is a distribution over vocabulary, and there are in total *K* topics. The generative process is a two-step process. First, we draw the proportion of each topic assignment, $z_{d,n}$ conditional on *d*. To include topic correlation, we drop the Dirichlet distribution and draw *d* from a logistic normal distribution^63^, i.e. $d \sim {\text {LogisticNormal}}(\mu , \Sigma )$. It is the covariance structure brought about by parameter that allows topic proportions to be correlated at the document-level. The key insight of this approach is then to map the real-valued vectors from the logistic-normal onto the simplex to recover a topic assignment in the form of a proportion, that is, $z_{d,n} ~ {\text {Multinomial(d)}}$. Then, as usual, we draw the proportions of each word $w_{d,n}$ in topics, conditional on topics assignment, $z_{d,n}$ , and topic k. The topic-word distribution corresponds to $w_{d,n} \sim {\text {Multinomial}}(d,k=z_{d,n})$. For more details, we refer the reader to the original paper by Blei and Lafferty^37^.

### Measuring a species interaction network

We follow Mariani^49^ in referring to the row-nodes as our ”active species”, in this case our social communities, while the column-nodes are the “passive species”, here topics. For simplicity, we refer to the former as community-nodes, and the later as topic-nodes. Then, each cell represents how much a community discusses a particular topic.

NODF is an overlap metric that calculates nestedness by first rearranging the interaction matrix of interest such that most interactions occupy the upper-left corner of the matrix (by decreasing fill, as in Fig. 2), then calculating the nested overlap^64^. We say that for any given pair of community-nodes (*i*, *j*), we have that most members of community *j* visit the same topics than community *i* if the degree of community *i* is larger than community j, or $c_i>c_j$. Then, we define the common neighbors of community *i* and *j* as $O_{ij} = A_iA_j$, that is, the sum of topic-node where the adjacency matrices *A* of both communities overlap. We write for community-NODF:

$$\begin{aligned} N_{C o m}=\sum _{(i, j)} \frac{O_{i j}}{c_{j}} \Theta \left( c_{i}-c_{j}\right) , \end{aligned}$$

where $\Theta$ is the so-called Heaviside function, e.g. a step function where $\Theta (x) = 1$ if $x > 0$ and $\Theta (x) = 0$ if $x = 0$. Practically, this means that if community j visits as many topics as community i, the value drops to zero. If $c_i > c_j$, then community-NODF is the common neighborhood over the degree of community j, which give us a percentage of overlap (with maximum nestedness when $O_{ij} = c_j$). If we do the same for topic-nodes, then we obtain degree of nestedness for the whole matrix:

$$\begin{aligned} \text {NODF} = \frac{N_{\text{ com } }+N_{\text{ topic } }}{\left[ \frac{n(n-1)}{2}\right] +\left[ \frac{m(m-1)}{2}\right] }, \end{aligned}$$

where the numerator is the sum of community- and topic-NODF and the denominator is the sum of $\frac{n(n1)}{2}$ row-nodes and $\frac{m(m1)}{2}$ column-nodes. High nestedness implies that we have something like an isocline running from the bottom-left corner to the upper right corner, whereby most interactions find themselves above it.

To detect modules in our network we maximize the Barber’s modularity *Q*^65^. To take into consideration the bipartite and weighted network of our network, we make use of the QuanBiMo algorithm^34,66^:

$$\begin{aligned} Q=\frac{1}{2 m} \sum _{i j}\left( A_{i j}-M_{i j}\right) \delta \left( c_{i}, c_{j}\right) , \end{aligned}$$

where $M_{ij}$ is a null model and is the indicator function that signals if a species belongs to a particular module or not, e.g. $(c_i,c_j) = 1$ if $c_i = c_j$ and 0 otherwise (see Barber 2007 for more details). The main difference from the original formula is that both $A_{ij}$ and $M_{ij}$ are weighted instead of binary. As mentioned above, in mutualistic networks we expect moderate modularity whereas modules are not strictly compartmentalized, but nonetheless exhibit significant modules. As modularity *Q* seeks to find clusters of interactions where within-module interactions are more prevalent than between-module interactions, we interpret modules here as joint socio-semantic (meta)communities whereby groups of communities and topical content interact more often than across the entire network.

## Supplementary Information

Supplementary Information.
