# Supplementary material for: Socio-semantic networks as mutualistic networks
Source: Sci Rep. 2022 Feb 3;12:1889. doi: 10.1038/s41598-022-05743-5 (PMC8813919; doi:10.1038/s41598-022-05743-5)
Supplement: Supplementary file 1 — Supplementary Information. [file 41598_2022_5743_MOESM1_ESM.pdf]

Supplementary Information for
"Socio-semantic networks as mutualistic networks"

Jonathan St-Onge, Louis Renaud-Desjardins,
Pierre Mongeau, Johanne Saint-Charles

I Data Preprocessing

The original Enron dataset is over 500,000 emails exchanges belonging to different 158 users[1]. To extract the original content sent by the core employees, we ignored emails addressed to oneself (e.g. self-loops) and we kept only the emails sent between the employees. This means that we do not count receivers listed in carbon copy and blind carbon copy as part of the exchanges. It is known that in the raw dataset, one employee may have different email addresses. This particular issue has already been addressed in the version we used hosted by Arne Hendrik Ruhe (http://www.ahschulz.de/enron-email-data/).

As mentioned in the main text, we focus on the email exchanges from March 1999 to February 2002, which correspond to the whole period of Enron activity(see Fig. S1 for a timeseries of the number of email exchanged during that period). Of the 500,000 emails in the original dataset, we are left with a social network of 44,638 links, and a corpus of 16,032 emails, or documents. In both cases, we have a total of 148 unique authors/nodes.

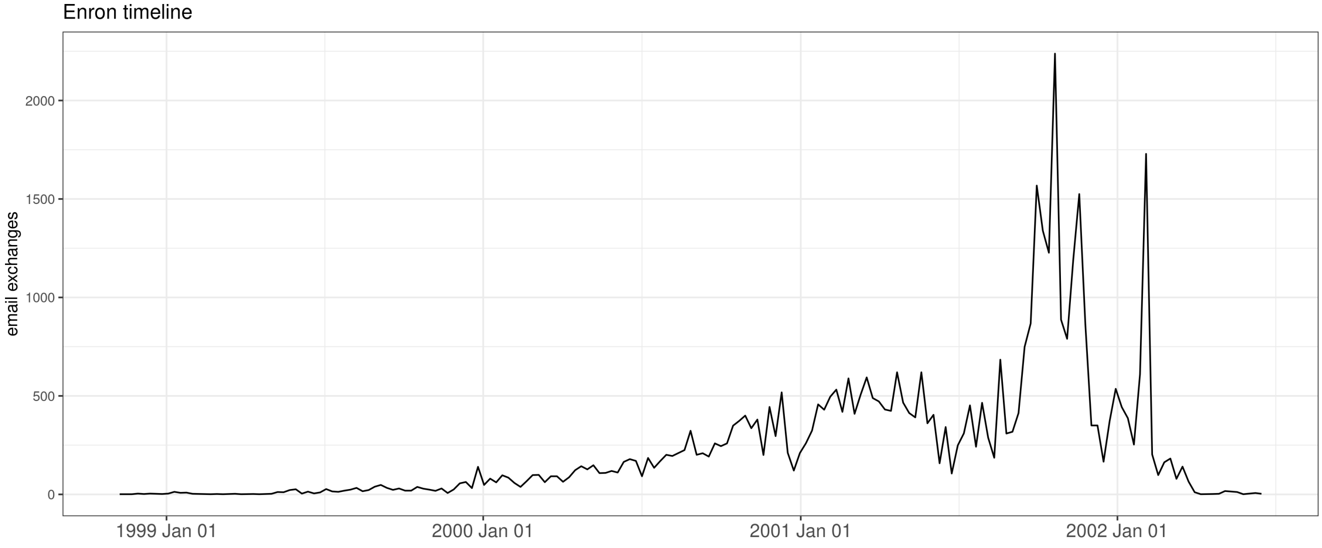

Figure S1. Enron timeline.

A key preprocessing step in our analysis that we haven't seen elsewhere is to keep only the original text written by the sender. This involves removing all replies in the text found after the original message. This cleaning step is in addition to the elimination of various boilerplate texts such as headers, email addresses and in-text signatures. Next, we have removed all numbers, dates, punctuation marks, words that appear fewer than five times. We used the snowball lexicon to remove stop words (http://snowball.tartarus.org/algorithms/english/stop.txt), we put the text in lowercase, as well as fixed encoding errors. We also lemmatized the corpus to improve the sparsity of our vocabulary. After cleaning, the dataset contains 14,470 emails, for a total of 38,312 words (or tokens) and a vocabulary of 7,234 unique words (or types).

II Methods

Stochastic Block Modeling

The Stochastic Block Model (SBM) assumes that all vertices in a graph G belong to a particular block B, where $b_i \in 1, ..., B$ and index i specifies node membership, and that vertices in the same blocks should interact more frequently than they interact with other blocks[2]. Thus, the probability that two vertices interact is only a function of their group assignment, denoted $w_{b_i b_j}$, whereby each vertex is assigned to only one block. An important advance in this field of research was to allow distribution of heterogeneous degrees. Whereas the traditional SBM expects that all vertices in a group have the same expected degree, the degree-corrected SBM (dcSBM) adds another parameter, i, that controls the expected degree for each node i, without regard for group membership[3].

As is the case with modularity optimization methods, the basic SBM suffers from a resolution limit[4]. As networks grow, it becomes impossible to find smaller communities, even if we are aware of their existence[5]. In addition, as it is the case with other popular community detection methods, SBMs must have the number of blocks specified in advance. To overcome these issues, as well as to avoid underfitting, Peixoto[?,6] recently developed a non-parametric, multilevel version of dcSBM—the nested dcSBM (ndcSBMs)—powered by an agglomerative multilevel Markov chain Monte Carlo algorithm[6].

Note that in our social network model, we have email counts as edge weights. This is easily handled by the ndcSBM by specifying the type of distribution we expect over edges, conditioned on the node partition. Since email exchanges show a positive count with no upper bound, as well as many people that do not exchange emails, we use the geometric distribution to model edge weights.

As for model selection, we follow Peixoto[7] and determine the quality of our models with the minimum description length (MDL). In a nutshell, the description length of a network is the amount of information we need to encode the observed pairs of interactions, given the generative structure we are assuming. The MDL principle states that among the models that have the same explanatory power, we must favor the one that is most capable of compressing information on the network. Practically, this means that we first calculate the entropy of a first model, H_{M1}, over the entropy of a second model, H_{M2}, what is referred to as the posterior log-odds ratio, and then we interpret a number greater than 1 as favoring the first model by a certain amount. This approach proves to be an effective means to assess over-fitting, that is, the tendency of our model to take random fluctuations in the data for an underlying structure[7].

To recapitulate, we take the advice of Fortunato and Hric[4] and we prefer the ndcSBM over other community detection methods for (i) its ability to find smaller communities (going from n to log n), via a multilevel strategy, which in turn has the advantage that we do not need to specify beforehand the right number of communities, (ii) its principled way to fight overfitting through minimisation of code description length, thereby mitigating the risk of finding non-existent partitions, and (iii) its versatility, allowing us to easily add edge covariates into the model that are weakly informative.

The Correlated Topic Model

To find topics in the Enron email corpus, we use the Correlated Topic Model (CTM) via the STM library[8] in the R programming language. We favor the STM library developped by Roberts, Stewart, and Tingley because it does not only provide a very efficient Expectation-Maximization algorithm to perform inference, but also a suite of tools to validate and visualize topics. Although the Structural Topic Model is the core model proposed by the library, the authors mention that the STM reduce to the CTM when no covariates are used. We favor the Correlated Topic Model over the Latent Dirichlet Allocation because it makes use of correlation to assess topics in a network, which has been shown to confer the CTM a predictive advantage[9,10].

Moreover, we find that the possibility to assess topic correlation in the form of a graph helps validating the model (Fig S2 bottom). For instance, we can see that topic 29, 8, 38, and 16 forms a clique. When we take a closer look, we can see that that they are topics related to a well-known issues between Enron and California (topic 38: power, energy, california, crisis) and what we could infer as their concern for the corporate image (topic 29: power, news, dow, court). Interestingly, these two topics are connected to the FERC investigation on their fraudulent activities (topic 16: ferc market, order, refunds). Taken all together, this give us a qualitatively validate our topics.

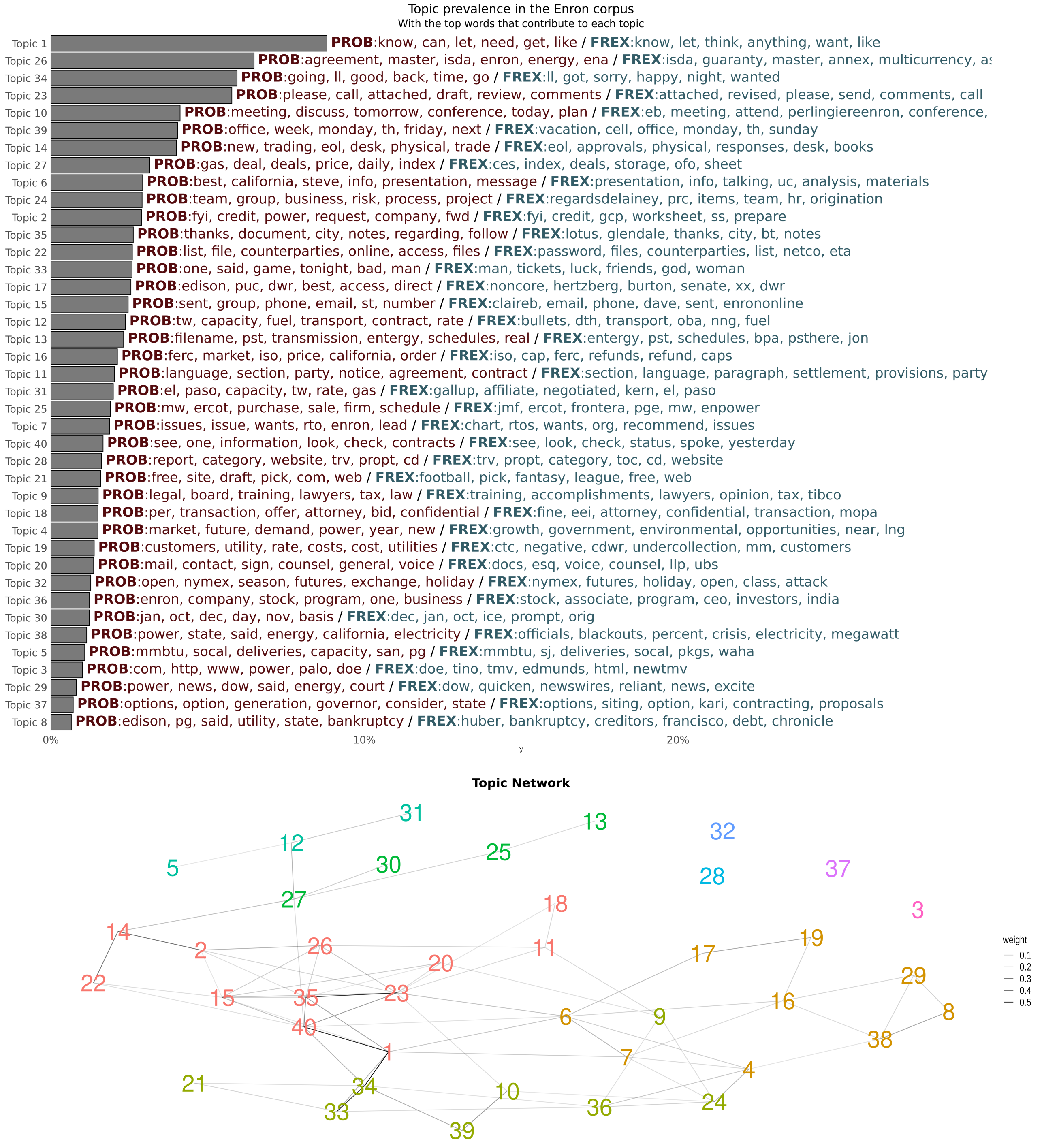

Figure S2. Enron semantic summary. Top: Enron topics with their most probable and exclusive words. On the x-axis, we have the prevalence of subjects, as indicated by the parameter γ. For each topic, we show the most probable words (in dark red), as well as the most exclusive words (in dark green), according to the FREX score. As there are many abbreviations, we have provided a glossary below. Bottom: Enron topic Network. Each node is a topic, and two topics are connected if they correlate above a threshold of 0.05. Strongly related topics mean that they often coocur in documents.

In contrast to ndcSBM, we explicitly use model selection to determine the relevant number of topics. To do so, we make use of held-out likelihood[11] and aim to strike a balance between topical semantic coherence and exclusivity[10,12]. The held-out likelihood is a common procedure for model selection whereby we remove a certain fraction of words within a document, which the model must then predict. We keep the default proposed by the STM library, and delete about half of the words in 10% from the document. In this scenario, we will prefer the number of topics that is most able to correctly predict this missing fraction of the corpus. Then, we want to strike a balance between topics that are both semantically coherent, which is a measure that has been shown to correlate with human judgment of topic quality, and the exclusivity of words to topic[13]. This model validation procedure led us to choose $K = 40$ topics, shown in the figure S2.

Although we recognize some limitations of the CTM as proxy for discourses, such as its inability to identify more sophisticated claim patterns[14] or to represent empirical properties for the frequency of words[15], we prefer it because it is a well-known model and its output are highly interpretable.

III Building the interaction matrix

We note two main differences between our interaction matrix and a true plant-pollinator matrix. First, both our axes are constructed from latent models. Hence, they have a certain number of assumptions that we must take into consideration when building the matrix. Most notably, because of the multinomial distribution over topic proportions, the CTM assumes that all topics are discussed in each document. This is obviouslyy wrong and has unwanted effects. The fact that CTM assumes that all documents contain scraps of all topics accumulates, and gives the false impression that some communities are talking about topics when they are not. To circumvent this issue, we first interpret all topics under 10% in a document as noise and replace them with zeros, and once we have summed over , all cells that did not sum up to three are also replaced with zeros. Filtering out below these thresholds amounts to "cut above the elbow" of the distributions, keeping only topics we can definitively observe in messages. Then, to calculate the interaction between topics and communities, we sum over all the for each document sent by a member of the community. In other words, the extent to which a community visits a topic is simply the sum of all the passages devoted to each topic within each email (after filtering) sent by the identified members. Second, the interactions in our interaction matrix are not counts but real numbers. We do not see this difference as a major impediment and we simply interpret the interactions as a continuous measure instead of a discrete value.

Quantifying mutualist networks

One issue is that when calculating nestedness with NODF, most tools target the topology of the network instead of its weighted counterpart. In this particular case, we are converting our weighted matrix to a binary matrix to use the tools at our disposal. Considering the aforementioned filtering steps where we are already getting rid of the smaller interaction values, this simplification at this point in the process does not make a significant change to the NODF output.

Comparing Enron interaction matrix to web-of-life mutualistic networks

To compare the Enron interaction matrix indices to true ecological networks, we uploaded all the data on pollinator and seed dispersal networks from Web-of-life ecological networks database (http://www.web-of-life.es/). We calculate all the different properties assumed to be characteristics of mutualistic networks mentioned in the main text for each of them (see Fig. S3). We then compare the average values with the Enron observed values. We find that all observed values calculated on the Enron interaction matrix fits comfortably within the bound of true ecological networks.

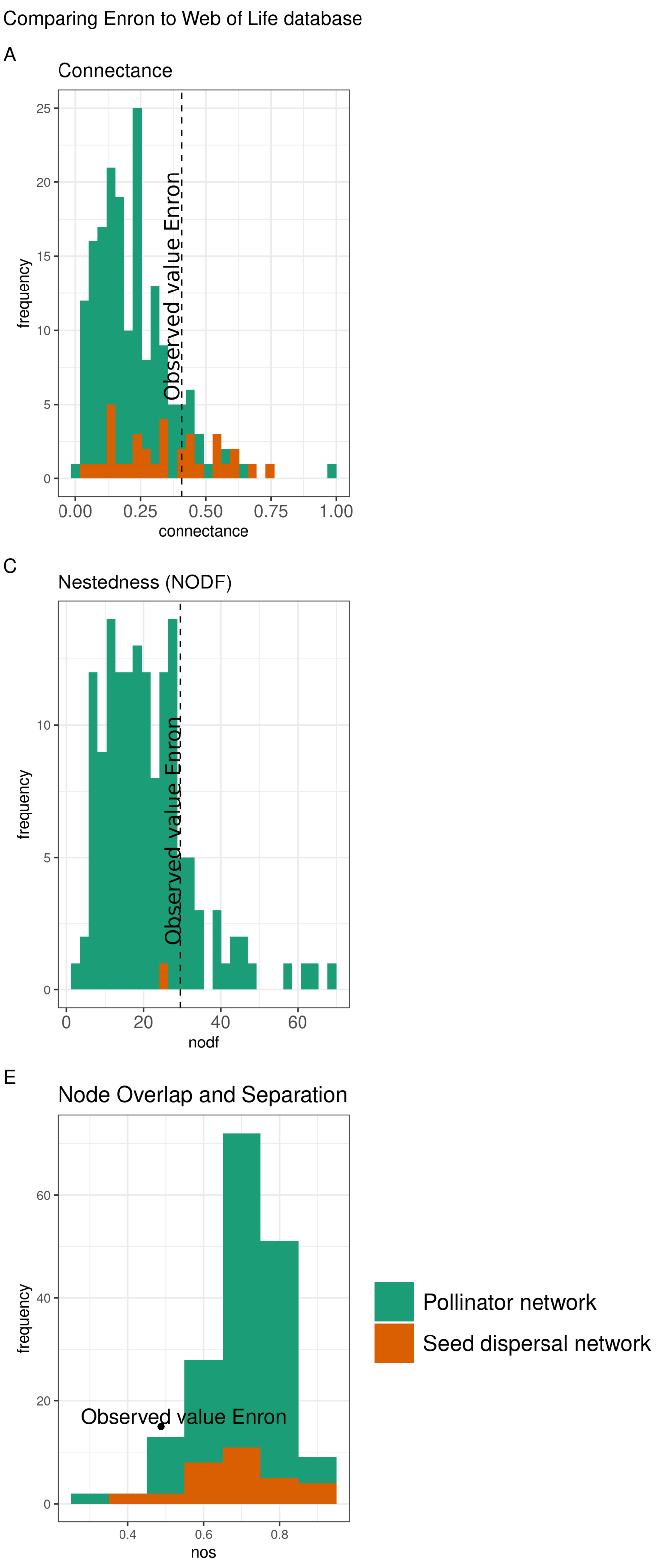

Figure S3. Comparing enron to Web of Life database. We compare enron's observed values with the same values for plant pollinators and seed dispersal networks in the Web of Life database. We note that in each case, enron sits comfortably within the limits of mutualist networks.

Table S1. Enron Lexicon

Abrev	Definition
1140	present in California Capacity Reports
28x	reference to senate bill 28x
80,000,000	reference to fraud money
ab1x	Assembly Bill
adc	american distribution company
aep	American Electric Power
agua	agua dulce, ville de Californie
alabamodome	stade football
anngtc	Alaskan Northwest Natural Gas Transportation Company
apex	apex-deposition
arem	Alliance for Retail Energy Markets
ares	alternative retail electric suppliers
ati	AirTouch Communications
brm	Business Relationship Management
calpeak	CalPeak Power LLC
cand	candidats
capex	capital expenses
cdwr	California Department of Water Resources
cec	California Energy Commission
ces	columbia energy services corporation
ces	combined energy service
cgas	California Gas Transport Inc
chkpf	stock market related
cinnabar	Cinnabar Energy, company
cng	compressed natural gas
cof	Capital One Financial Corp. (stock market)
cows	use to explain economy (capitalism vs communism)
crestar	Crestar Energy, company
cpuc	California Public Utilities Comission
csco	stock market related
csfb	Swiss Credit First Boston
ctc	competitive transition charge
da	spanish
dasr	de-DASR, reference to a conflict with UC/CSU, University of California
dart	project, product, deals
dash	reference to fantasy football
dedasr	de-DASR, reference to a conflict with UC/CSU, University of California
dh	finance stock market
dth	dekatherm = 1 mmbtu
dtn	data transmission network
dulce	agua dulce, city in California
dwr	California Department of Water Resources
eci	enron credit inc
edi	EDI hub, company Connecting to Trading Partners
edison	Edison Electric Institute
eei	Edison Electric Institute
egas	Enron Global Assets and Services
eisb	electrical industry service bureau
emc	Dell ECM corporation (stock market)
eml	enron metal ltd.
eob	explanation of benefits
eol	end-of-line (annual maintenance)
ercot	electric reliability council of texas
esps	energy service providers
ets	ghost company

fb	stock market related
ferrous	reference to Cargill Metals Supply Chain
ft	feet
fsa	Financial Services Authority
gerrymanderi	gerrymandering
gisb	Gas Industry Standards Board
gj	giga joule
gmbh	company(s) subsidiaries Enron
gorons	political joke anti-Gore
hemu	email link company Dynergy
hesco	houston engineering services company, energy investment
iep	independant energy producer
iso	California Independent System Operator
ivanhoe	problem at ivanhoe station
lch	London Clearing House, compensation chamber
lvcii	Las Vegas Cogeneration LP II, energy company
lxk	word used by Option Investors (io)
negm	Northeast Gas Markets
mevco	company
mgmt	management
mmbtu	Million British Thermal Units
mou	memorandum of understanding
msft	microsoft, stock market
mu	Micron Technology, Inc.(stock market)
narg	north american regional gas
nful	National Fuel Gas Company
ng	natural gas
nggj	nggj gj cad, show up in stock market reports
nng	northern natural gas, bought by Enron then by Dynergy
OWS	Oil Field Warehouse & Services Limited
oi	option investor, site web
oex	false stock index invented by Option Investors (io)
piggi	method to clean oil pipelines
pjm	company that coordinate electricity sells
ptc	production tax credit
pub	Public Utilities Comission
puc	Public Utilities Comission
px	power exchange
RAC	Risk Assessment and Control division de Enron
roi	return on investment
rto	regional transmission organizations
sb	senate bill
sce	Southern California Edison
sme	society for mining, metallurgy and exploration
snc	stock market
sonat	energy company
sunw	sunwork, stock market
tdcc	the dow chemical company
tlab	Tellabs, Inc (stock market)
trco	Transco Energy Co LLC (Company)
transco	Transco Energy Co LLC (Company)
tribolet	michael tribolet, employé
tw	ghost company
usaee	United States Association for Energy Economics
ut	University of Texas
utx	United Technologies Corporation (stock market)
vix	stock market
vod	stock market company
wcom	stock market company
xirc	xircom
zima	drink

Bibliography

[1] Klimt, B. & Yang, Y. Introducing the Enron Corpus. 2.

[2] Holland, P. W., Laskey, K. B. & Leinhardt, S. Stochastic blockmodels: First steps. *Social Networks* **5**, 109–137, DOI: 10.1016/0378-8733(83)90021-7 (1983).

[3] Karrer, B. & Newman, M. E. J. Stochastic blockmodels and community structure in networks. *Physical Review E* **83**, 016107, DOI: 10.1103/PhysRevE.83.016107 (2011). ArXiv: 1008.3926.

[4] Hric, D., Peixoto, T. P. & Fortunato, S. Network Structure, Metadata, and the Prediction of Missing Nodes and Annotations. *Physical Review X* **6**, 031038, DOI: 10.1103/PhysRevX.6.031038 (2016).

[5] Fortunato, S. Community detection in graphs. *Physics Reports* **486**, 75–174, DOI: 10.1016/j.physrep.2009.11.002 (2010). ArXiv: 0906.0612.

[6] Peixoto, T. P. Efficient Monte Carlo and greedy heuristic for the inference of stochastic block models. *Physical Review E* **89**, 012804, DOI: 10.1103/PhysRevE.89.012804 (2014). ArXiv: 1310.4378.

[7] Peixoto, T. P. Model Selection and Hypothesis Testing for Large-Scale Network Models with Overlapping Groups. *Physical Review X* **5**, 011033, DOI: 10.1103/PhysRevX.5.011033 (2015).

[8] Roberts, M. E., Stewart, B. M. & Tingley, D. stm : An R Package for Structural Topic Models. *Journal of Statistical Software* **91**, DOI: 10.18637/jss.v091.i02 (2019).

[9] Blei, D. M. & Lafferty, J. D. A correlated topic model of Science. *The Annals of Applied Statistics* **1**, 17–35 (2007).

[10] Roberts, M. E., Stewart, B. M. & Airoldi, E. M. A Model of Text for Experimentation in the Social Sciences. *Journal of the American Statistical Association* **111**, 988–1003, DOI: 10.1080/01621459.2016.1141684 (2016).

[11] Wallach, H. M., Murray, I., Salakhutdinov, R. & Mimno, D. Evaluation methods for topic models. In *Proceedings of the 26th Annual International Conference on Machine Learning - ICML '09*, 1–8, DOI: 10.1145/1553374.1553515 (ACM Press, Montreal, Quebec, Canada, 2009).

[12] Bischof, J. M. & Airoldi, E. M. Summarizing topical content with word frequency and exclusivity. 8 (2012).

[13] Mimno, D., Wallach, H. M., Talley, E., Leenders, M. & McCallum, A. Optimizing Semantic Coherence in Topic Models. In *Proceedings of the Conference on Empirical Methods in Natural Language Processing*, EMNLP '11, 262–272 (Association for Computational Linguistics, Stroudsburg, PA, USA, 2011). Event-place: Edinburgh, United Kingdom.

[14] Menezes, T. & Roth, C. Semantic Hypergraphs. *arXiv:1908.10784 [cs]* (2019). ArXiv: 1908.10784.

[15] Gerlach, M., Peixoto, T. P. & Altmann, E. G. A network approach to topic models. *Science Advances* **4**, eaaq1360, DOI: 10.1126/sciadv.aaq1360 (2018).
